# Successful conversion surgery after comprehensive therapy in a patient with MSI-H but pMMR metastatic gastric mixed adenoneuroendocrine carcinoma: a case report and literature review

**DOI:** 10.3389/fonc.2024.1463884

**Published:** 2024-12-11

**Authors:** Jun He, Li Wang, Chuanlei Tuo, Weihua Gong, Yong Liu

**Affiliations:** ^1^ Department of Surgery, Second Affiliated Hospital of School of Medicine, Zhejiang University, Hangzhou, China; ^2^ Department of Emergency Medicine, Second Affiliated Hospital of School of Medicine, Zhejiang University, Hangzhou, China; ^3^ Department of Gastroenterology, Tianjin Medical University Cancer Institute and Hospital, National Clinical Research Center for Cancer, Tianjin, China; ^4^ Key Laboratory of Cancer Prevention and Therapy, Tianjin, China; ^5^ Tianjin’s Clinical Research Center for Cancer, Tianjin, China

**Keywords:** gastric mixed adenoneuroendocrine carcinoma, conversion surgery, targeted therapy, immunotherapy, chemotherapy

## Abstract

Gastric mixed adenoneuroendocrine carcinoma (MANEC) is a rare and highly aggressive malignancy characterized by both exocrine and neuroendocrine components. Treatment options for metastatic cases are limited, with typical therapeutic approaches involving a combination of chemotherapy and immunotherapy. A 68-year-old male with metastatic gastric MANEC was treated with targeted therapy, immunotherapy, and chemotherapy, including S-1, apatinib, cadonilimab, and paclitaxel. After six cycles, the liver metastases resolved completely, and the primary tumor achieved partial remission, leading to conversion surgery. The patient underwent a radical D2 gastrectomy with R0 resection, including proximal gastrectomy, splenectomy, omentectomy, and esophagogastric anastomosis, along with radiofrequency ablation of liver metastases. Postoperative pathology confirmed the disappearance of liver metastases but revealed residual adenocarcinoma in the primary gastric lesion and neuroendocrine components in the perigastric lymph nodes. The patient was discharged seven days post-surgery. Five months postoperatively, new liver metastases were detected, exhibiting neuroendocrine differentiation. The patient was subsequently treated with a maintenance regimen of S-1 and pembrolizumab. This case highlights the significant heterogeneity of gastric MANEC and the challenges in managing such cases. While conversion surgery can be effective in certain contexts, the high likelihood of postoperative recurrence and metastasis, particularly in neuroendocrine components, necessitates cautious consideration. Further research is needed to evaluate the long-term benefits of conversion surgery in metastatic gastric MANEC and to develop tailored therapeutic strategies.

## Introduction

Gastric mixed adenoneuroendocrine carcinoma (MANEC) is a rare type of gastric tumor characterized by both exocrine and neuroendocrine components, each comprising more than 30% of the tumor (1). In 2019, the World Health Organization (WHO) classified MANEC as part of the spectrum of gastric mixed neuroendocrine-non-neuroendocrine neoplasms (2). Gastric MANEC is more aggressive and more likely to metastasize distantly compared to gastric adenocarcinoma, making its treatment more challenging (3). Despite the lack of effective treatments for distant metastatic gastric MANEC, clinicians commonly employ chemotherapy and immunotherapy. However, this carcinoma typically shows poor responsiveness to chemotherapy (4). Retrospective studies have suggested that conversion surgery may confer a survival benefit for stage IV gastric adenocarcinoma patients (5–7). However, there is ongoing controversy regarding whether conversion surgery offers similar benefits for patients with stage IV gastric neuroendocrine carcinoma or gastric MANEC (8, 9).

In this report, we present a unique case of a patient with distant metastatic gastric MANEC exhibiting microsatellite instability-high (MSI-H) but proficient mismatch repair (pMMR). This patient underwent conversion surgery following a regimen of targeted therapy, immunotherapy, and chemotherapy. The conversion surgery was successful, achieving both a D2 gastrectomy and an R0 resection. Our aim is to contribute to a deeper understanding of the potential role of conversion surgery in the treatment of metastatic gastric MANEC.

## Case presentation

In March 2023, a 68-year-old male presented to a local hospital with intermittent upper abdominal pain for three months. The patient reported a weight loss of 3 kg over the past three months and mentioned occasional alcohol consumption and smoking for over forty years. The patient’s BMI at the time of presentation was 20.1. Gastroscopy revealed a large irregular lesion on the lesser curvature side of the gastric cardia and body, with pathology indicating moderately differentiated adenocarcinoma. The patient had no significant medical history and no comorbidities such as diabetes or hypertension. Moreover, the patient has no family history of tumors or hereditary diseases. For further evaluation and treatment, the patient was referred to our center.

**Figure 1:**
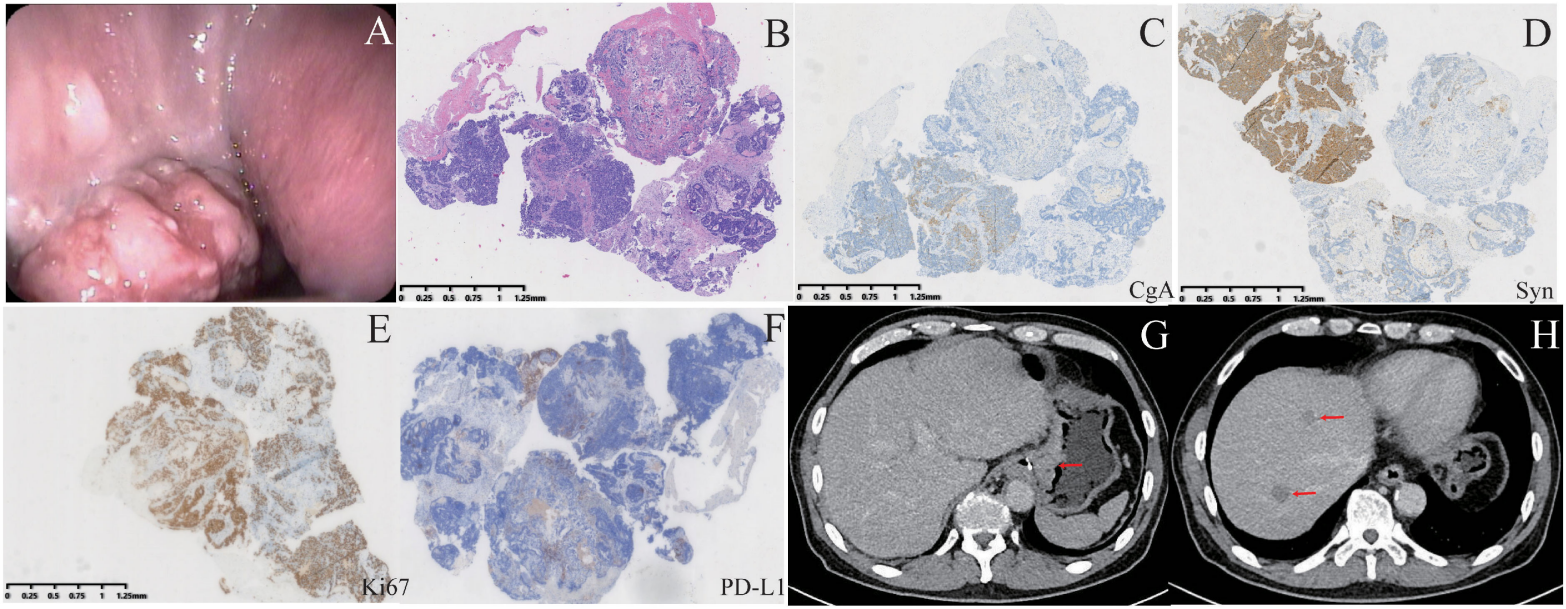
Patient examinations upon first admission. **(A)** A tumor is visible during gastroscopy. **(B)** HE staining of gastroscopic biopsy samples. **(C–F)** Immunohistochemical staining for CgA, Syn, Ki67, and PD-L1. CgA and PD-L1 exhibited focal positivity, whereas Syn and Ki67 showed strong positivity. **(G, H)** CT revealed gastric wall thickening and liver metastases.

At our center, a thorough physical examination was performed on the patient. The patient’s abdomen was flat, with mild tenderness in the left upper quadrant. No abnormalities were found in the physical examination of the rest of the body. Moreover, enhanced abdominal computed tomography (CT), gastric endoscopic ultrasonography, and immunohistochemical examination of gastric biopsies were performed (**Figure 1**). CT revealed thickening of the gastric wall at the cardia and fundus, extending into the lower esophagus, along with retroperitoneal lymph node metastasis. Additionally, multiple low-density nodules were identified in the liver, suggesting gastric cancer liver metastases. Pathological and immunohistochemical results suggested that the gastric tumor was a gastric mixed adenoneuroendocrine carcinoma. Additionally, the immunohistochemistry for PD-L1 of the gastric tumor cells indicated focal positivity. The gastric MANEC was classified as stage IV (cT4N3M1).

**Figure 2:**
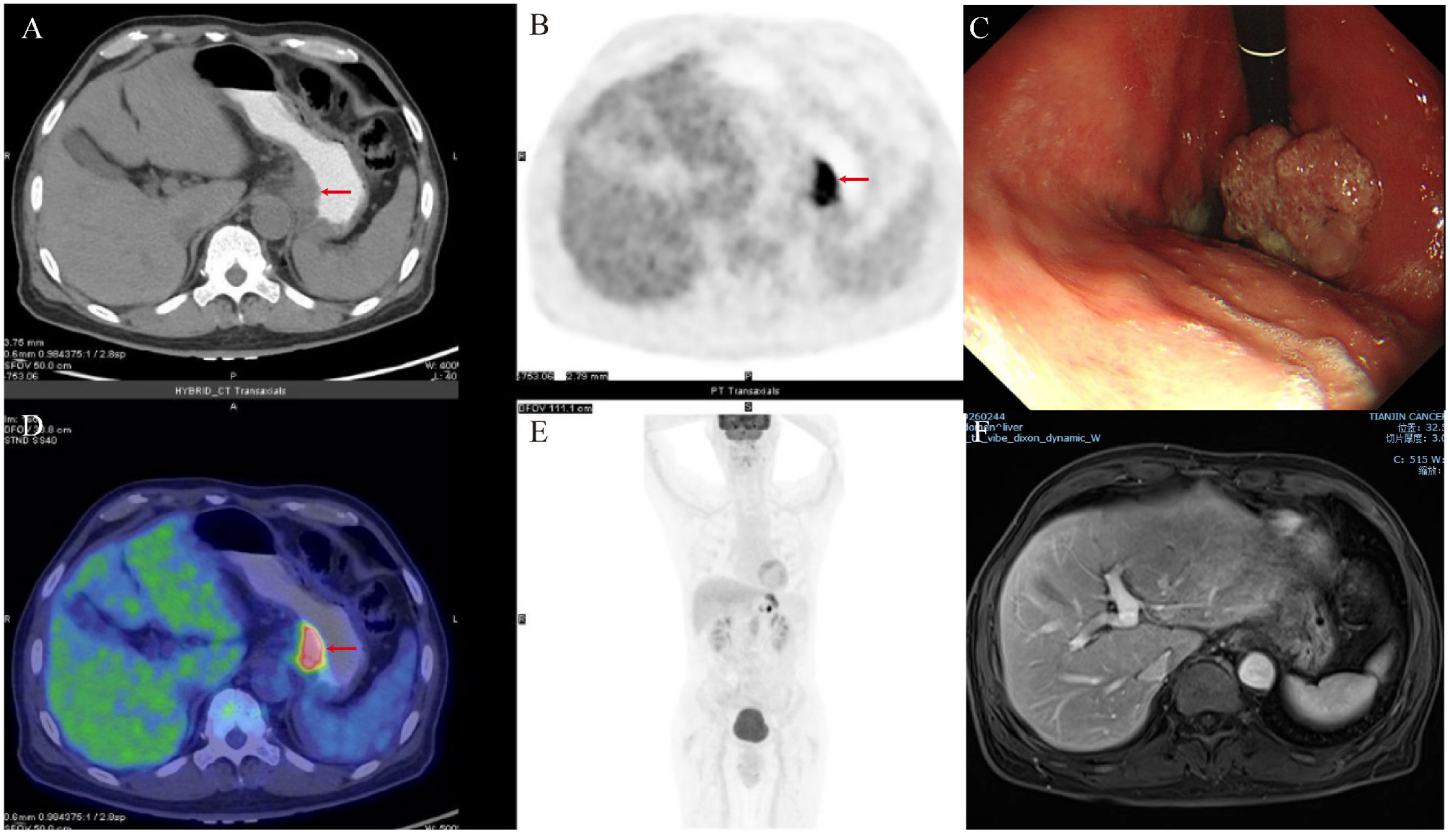
Examinations of the patient after comprehensive treatment. **(A, B, D, E)** Gastric tumors shrank, and liver metastases disappeared on PET−CT after neoadjuvant therapy. **(C)** The tumor volume showed reduction during gastroscopy. **(F)** MRI revealed the absence of liver metastases.

Due to liver metastasis from the tumor, a multidisciplinary team, including oncologists, gastroenterological surgeons, and radiologists, was convened to discuss the treatment for this patient. Ultimately, they decided on a treatment regimen comprising S-1 (60 mg, orally, twice daily on days 1 to 14), apatinib (0.25 g, orally, once daily on days 1 to 21), cadonilimab (720 mg, intravenously, on day 1), and paclitaxel (210 mg, intravenously, on day 1), with each treatment cycle lasting three weeks. After six treatment cycles, the primary tumor achieved partial remission, and the liver metastases disappeared (**Figure 2**). In the seventh cycle, apatinib was discontinued, and the patient continued with chemotherapy and immunotherapy, followed by conversion surgery. No comorbidities were found during the preoperative examination.

**Figure 3:**
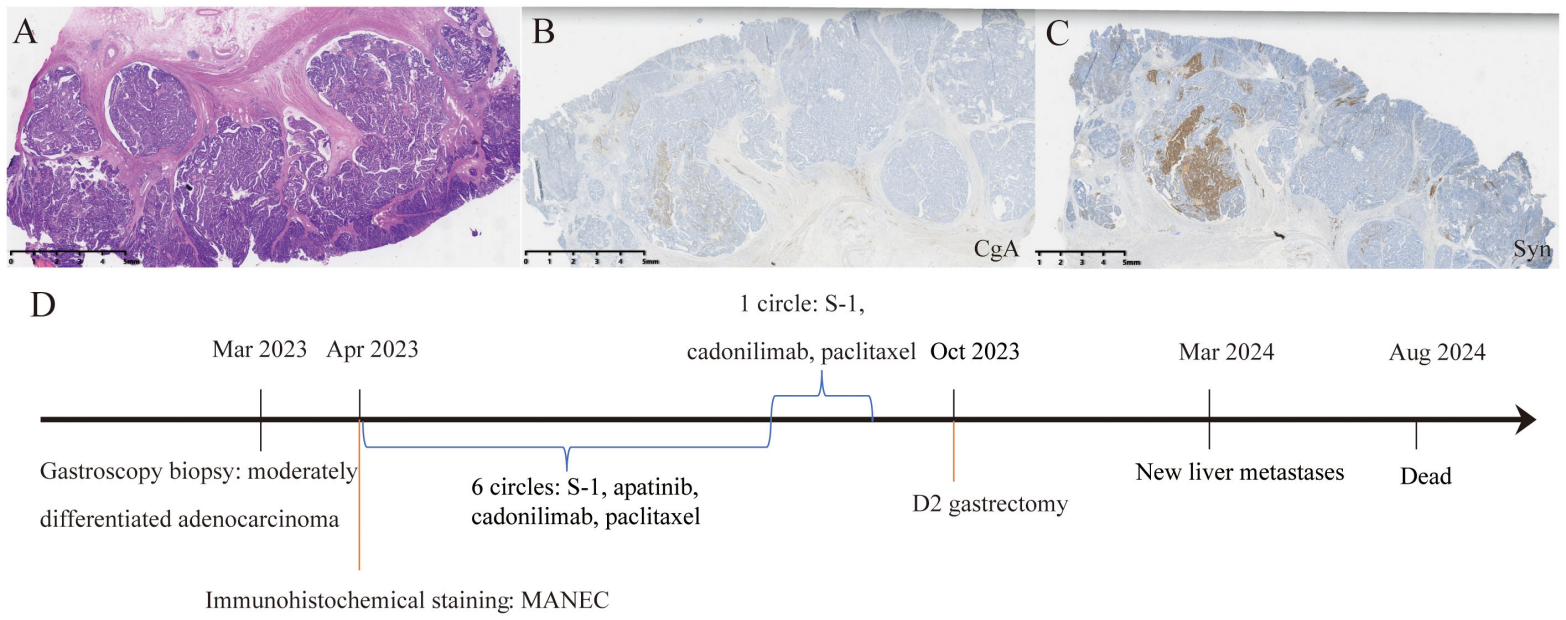
Treatment procedure and postoperative pathological examination of the gastric tumor. **(A)** HE staining of postoperative gastric tumor. **(B, C)** Immunohistochemical staining for CgA and Syn exhibited focal positivity. **(D)** The patient’s treatment course.

The surgery was performed via an open approach, lasting 312 minutes with a blood loss of 328ml. The procedure went smoothly, with no intraoperative or postoperative complications. The surgery involved proximal gastrectomy, splenectomy, omentectomy, and esophagogastric anastomosis, along with intraoperative frozen pathology assessment and radiofrequency ablation of the liver metastases. R0 resection was successfully achieved, and the patient recovered well, being discharged seven days after the operation. Intraoperative frozen pathology evaluation revealed inflammatory cells in the liver metastases. Postoperative pathological examination revealed a substantial amount of residual adenocarcinoma tissue in the primary gastric lesion, with a tumor regression grade (TRG AJCC 8th) of three (**Figure 3**). A total of 41 lymph nodes were retrieved during surgery, and two positive lymph nodes were detected. The pathological and immunohistochemical results of the gastric lesion confirmed it as gastric MANEC with pMMR. Additionally, the tumor was negative for both Epstein–Barr virus (EBV) and HER2. However, the polymerase chain reaction (PCR)-based genetic testing revealed that the gastric MANEC exhibited MSI-H. Subsequent genetic testing of multiple gastric lesions consistently indicated MSI-H status. After surgery, the gastric MANEC was classified as stage IIa (ypT2N1M0). The patient declined further adjuvant treatment due to financial reasons and opted for regular follow-up instead.

**Figure 4:**
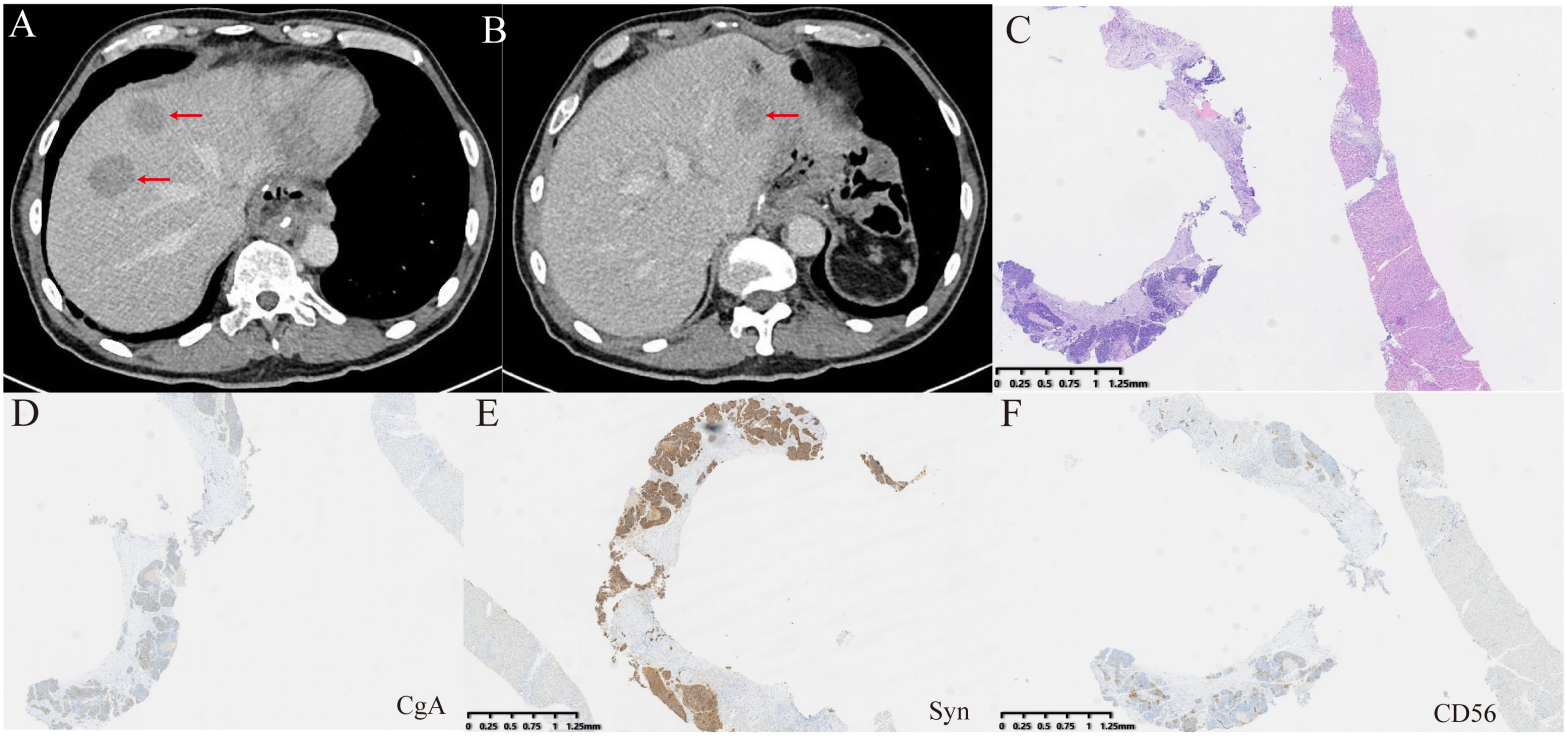
Examinations of liver metastases after surgery. **(A, B)** CT revealed new liver metastatic lesions. **(C)** HE staining was performed on the biopsy tissue from the new liver metastases. **(D–F)** Immunohistochemical staining for CgA, Syn, and CD56. CgA and CD56 exhibited focal positivity, while Syn showed strong positivity.

Five months after surgery, the patient returned for follow-up. Abdominal CT revealed multiple new low-density lesions in the liver (**Figure 4**). Subsequently, a biopsy of the lesions confirmed poorly differentiated carcinoma with a neuroendocrine component. Based on the patient’s medical history, these findings were suggestive of tumor recurrence. Due to the patient’s poor general condition and advanced age following tumor recurrence, S-1 monotherapy was chosen for chemotherapy. Additionally, as the tumor was MSI-H, which tends to respond well to immunotherapy (PD-1 inhibitor), pembrolizumab was selected. Despite treatment with S-1 and pembrolizumab, the disease continued to progress. Given the patient’s poor general condition and severely impaired liver function, palliative care was chosen for subsequent management. The patient passed away in August of this year, with an overall survival of 17 months.

## Discussion

Gastric MANEC, as a rare malignant tumor, exhibits biological behavior different from that of gastric adenocarcinoma and tends to metastasize distantly at an early stage (10). A study has found that when the neuroendocrine component in gastric cancer exceeds 10%, it becomes an unfavorable prognostic factor (11). For early-stage gastric neuroendocrine carcinoma and gastric MANEC, surgery is the primary curative approach. However, once gastric MANEC metastasizes, chemotherapy becomes the mainstay of treatment, and surgery is generally not considered. In cases of severe complications such as bleeding or pyloric obstruction, palliative resection may be performed.

Conversion surgery involves performing radical surgery on both primary and metastatic lesions following partial or complete tumor remission achieved through chemotherapy, targeted therapy, immunotherapy, or other treatments. Patients with initially metastatic gastric adenocarcinoma may experience improved survival outcomes if they undergo conversion surgery and achieve R0 resection following chemotherapy. Furthermore, several case reports document successful conversion surgery to achieve a pathological complete response in metastatic gastric adenocarcinoma through the combination of chemotherapy and immunotherapy (12, 13). Indeed, some retrospective studies have indicated that the primary tumor surgery might offer survival advantages to patients with metastatic gastric cancer (14–16). However, it is important to note that these studies included various types of gastric cancer and did not specifically focus on subgroups such as gastric neuroendocrine carcinoma or gastric MANEC. Research on the application of conversion surgery for metastatic gastric MANEC is very limited. As treatment options for metastatic gastric MANEC expand, the feasibility of conversion surgery remains uncertain when tumors achieve partial or complete remission following various systemic treatments.

Microsatellite instability (MSI) is a crucial molecular subtype of gastric cancer that can guide therapeutic decisions. Typically, MSI-H in gastric cancer is closely associated with deficient mismatch repair (dMMR) (17, 18). Interestingly, genetic testing revealed that the gastric MANEC in this patient exhibited MSI-H despite the tumor’s pMMR status. Additional testing is recommended when results inconsistent with MSI-H and dMMR are found in gastric cancer. Therefore, we conducted genetic testing on multiple gastric lesions post-surgery, as well as on the lesions obtained during the initial gastroscopy. The results consistently indicated MSI-H status. The interpretation of the immunohistochemistry results for MMR proteins may also be the reason for this discrepancy (19). Independent reviews by different pathologists consistently confirmed that the tumor exhibited pMMR based on immunohistochemical findings. In a study comparing MSI testing and MMR protein immunohistochemistry, which analyzed over 5,000 gastric cancer tissue samples, the occurrence rate of MSI-H with pMMR was only 0.05% (20). Rare missense mutations in MMR proteins, such as MLH1 and MSH6, that impact protein function without affecting protein translation or antigenicity, may contribute to this discrepancy (21, 22). Next-generation sequencing can be employed to detect these missense mutations. Moreover, it is probably due to alterations in other pathways, mutations in specific regions of the genome that cause microsatellite instability without directly altering the proteins responsible for mismatch repair. Finally, the technical limitations of immunohistochemistry tests may also be one of the reasons.

The survival benefit of chemotherapy in patients with dMMR/MSI-H gastric cancer remains controversial (23). The large number of mutations in MSI-H tumors generate neoantigens, which activate the immune system, resulting in a better response to immunotherapy (24, 25). Notably, research has demonstrated that dual immunotherapy (nivolumab plus ipilimumab) targeting PD-1 and CTLA-4 in locally advanced dMMR/MSI-H gastric or gastroesophageal adenocarcinoma cases can achieve a pathological complete response (pCR) in over half of the patients (24). In our study, the immunohistochemistry for PD-L1 in gastric tumor cells revealed focal positivity, suggesting that the patient is likely to benefit from immunotherapy. We employed a novel immunotherapy drug called cadonilimab. Cadonilimab represents the world’s first bispecific antibody targeting both PD-1 and CTLA-4 (26). In 2017, pembrolizumab received approval from the U.S. Food and Drug Administration (FDA) for unresectable or metastatic dMMR/MSI-H solid tumors (27). Postoperatively, with the emergence of new liver metastases, the patient received combination therapy with pembrolizumab and S-1.

Before the surgery, we adopted a multi-drug regimen based on the recommendations from the multidisciplinary team. The oncologists proposed the use of apatinib for anti-angiogenesis, as it is a targeted therapy approved for gastric cancer, alongside trastuzumab. Cadonilimab has been shown in studies to improve survival outcomes in patients with advanced gastric cancer, including those with low PD-L1 expression. Given the significant side effects associated with platinum-based chemotherapy, along with the patient’s age and the use of multiple medications, we opted for a chemotherapy regimen combining S-1 and paclitaxel. The radiologists recommended against radiotherapy due to the presence of multiple metastatic lesions in the liver, which made it unsuitable for treatment at this stage. The treatment plan was adjusted based on the patient’s response, and due to the favorable outcome observed prior to surgery, no alterations were made to the treatment strategy. Moreover, the patient did not experience significant symptoms from apatinib. Regarding chemotherapy with S-1 and paclitaxel, the patient reported mild fatigue and occasional nausea, which were managed with symptomatic treatment. As for immunotherapy with cadonilimab, the patient did not experience any significant immune-related adverse events.

Throughout the course of treatment, the patient demonstrated excellent cooperation. However, upon initially being informed of the liver metastasis, the patient lost confidence in the treatment. With encouragement from both the family and the medical team, along with the positive effects of comprehensive treatment, the patient regained motivation for therapy. We fully understand the patient’s decision to forgo adjuvant therapy after surgery due to financial constraints. Following postoperative recurrence and progression, with limited efficacy from further treatment and considering the patient’s suffering, we recommended palliative care to alleviate the patient’s discomfort.

Although the liver metastasis probably disappeared after preoperative treatment, it is still possible that cancer cells have metastasized to other parts of the body but were not detected. The early postoperative liver lesions may have originated from cancer cells that had metastasized to other sites before surgery. Notably, both lymph node and liver metastases following systemic therapy were composed of neuroendocrine components. This observation indicates that neuroendocrine components in gastric cancer exhibit greater malignancy and a higher likelihood of metastasis. Research has found that the neuroendocrine component in gastric MANEC originates from adenocarcinoma, leading to increased malignancy (28). In the few reported cases of metastatic gastric MANEC undergoing surgery, new liver metastases often developed shortly afterward (**Table 1**). In this study, new liver metastases were discovered five months post-surgery and identified as neuroendocrine carcinoma. Although previous reports did not biopsy the new liver metastases, it is highly likely they were also neuroendocrine carcinoma, given the tumor’s propensity for metastasis and recurrence. Despite achieving R0 resection, conversion surgery did not improve survival in this patient. This may be closely related to the neuroendocrine component of the tumor.

**Table 1:** Summary of surgical treatment cases for metastatic gastric mixed adenoneuroendocrine carcinoma.

Author (year)	Age/gender	Site of gastric tumor	Site of organ metastasis	Component of the metastasis	Preoperative treatment	Surgical method	Postoperative treatment	Site of recurrence	Time to recurrence (month)
Chen et.al, 2019 (29)	59/Male	Fundus, cardia	Unkonwn	Unkonwn	Chemotherapy and radiology	Palliative gastrectomy	Unkonwn	Liver	12
Nagata et.al, 2020 (30)	60/Male	Greater curvature, the upper corpus	Liver	Unkonwn	Chemotherapy	Total gastrectomy distal pancreatosplenectomy and extended left hepatic lobectomy	Chemotherapy	Liver	3
Zhang et al., 2014 (31)	68/Male	Antrum	Liver	Neuroendocrine carcinoma	None	Left hepatectomy and distal gastrectomy	None	Liver	2.5
Inaba et al., 2017 (32)	61/Male	Anterior wall of pylorus	Liver	Unkonwn	Chemotherapy	Distal gastrectomy and liver metastasectomy	Chemotherapy	Liver	2

These findings highlight the significant heterogeneity of gastric cancer. While conversion surgery can yield favorable outcomes for advanced gastric adenocarcinoma in the era of targeted and immunotherapy, caution should be exercised when considering conversion surgery for advanced gastric neuroendocrine carcinoma and mixed adenoneuroendocrine carcinoma. Further research is needed to evaluate the long-term benefits of conversion surgery in metastatic gastric MANEC and to develop tailored therapeutic strategies.

## Conclusion

This case demonstrates the potential benefit of conversion surgery for metastatic gastric MANEC following systemic therapy, including targeted therapy, immunotherapy, and chemotherapy. Although the conversion surgery achieved an R0 resection, the recurrence of neuroendocrine-differentiated liver metastases highlights the aggressive nature of the disease. These findings suggest that while conversion surgery can be effective for advanced gastric adenocarcinoma, it requires careful consideration for gastric MANEC. Further research is essential to evaluate the long-term outcomes and develop tailored therapeutic strategies for metastatic gastric MANEC.

## Data Availability

The original contributions presented in the study are included in the article/supplementary material. Further inquiries can be directed to the corresponding author.
